# Supplementary material for: Molecular Characterization of Staphylococcus aureus Isolated from Raw Milk Samples of Dairy Cows in Manhiça District, Southern Mozambique
Source: Microorganisms. 2021 Aug 8;9(8):1684. doi: 10.3390/microorganisms9081684 (PMC8402116; doi:10.3390/microorganisms9081684)
Supplement: Supplementary file 1 [file microorganisms-09-01684-s001.zip › microorganisms-1297422-SI/microorganisms-1297422 supplementary/Table S1.pdf]

Table S1. Primers used in this study

Target Gene	Primers	Nucleotide Sequence (5′-3′)	Amplicon Size (bp)	Reference
S. aureus **identification**				
nuc	pnuc_Fw	GCGATTGATGGTGATACGGTT	270	[1]
	pnuc_Rv	AGCCAAGCCTTGACGAACTAAAGC		
**Screening of resistance determinants**				
mecA	mecA_Fw	GGTCCCATTAACTCTGAAG	1040	[2]
	mecA_Rv	AGTTCTGCAGTACCGGATTTGC		
blaZ	blaZ_Fw	GATAAGAGATTTGCCTATGC	533	[3]
	blaZ_Rv	GCATATGTTATTGCTTGACC		
erm(A)	erm(A)_Fw	AAGCGGTAAACCCCTCTGAG	442	[4]
	erm(A)_Rv	AAGCGGTAAACCCCTCTGAG		
erm(C)	erm(C)_Fw	TCGTAACTGCCATTGAAATA	348	[5]
	erm(C)_Rv	TCACTTTAGGTTTAGGATGAAA		
msr(A)	msr(A)_Fw	GATTGTCCCAAGCCAGTAAA	445	[6]
	msr(A)_Rv	GCCATTTGCACTTTAGGAGA		
tet(K)	tet(K)_Fw	GTAGCGACAATAGGTAATAGT	361	[7]
	tet(K)_Rv	GTAGTGACAATAAACCTCCTA		
tet(M)	tet(M)_Fw	GTTAAATAGTGTTCTTGGAG	657	[8]
	tet(M)_Rv	CTAAGATATGGCTCTAACAA		
tet(L)	tet(L)_Fw	GTCGGTAATTGGGTTTGTTG	421	[9]
	tet(L)_Rv	TGACAGCACGCTAACGATAA		
dfrG	dfrG_Fw	TTTCTTTGATTGCTGCGATG	501	[10]
	dfrG_Rv	AACGCACCCGTTAACTCAAT		
dfrA(S1)	dfrA(S1)_Fw	CACTTGTAATGGCACGGAAA	270	[11]
	dfrA(S1)_Rv	CGAATGTGTATGGTGGAAAG		
**Virulence genes**				
sea	sea-Fw	ACGATCAATTTTTACAGC	544	[12]
	sea-Rv	TGCATGTTTTCAGAGTTAATC		
seb	seb-Fw	ATTCTATTAAGGACACTAAGTTAGGGA	404	[12]
	seb-Rv	ATCCCGTTTCATAAGGCGAGT		
sec	sec-Fw	GACATAAAAGCTAGGAATTT	257	[12]
	sec-Rv	AAATCGGATTAACATTATCCA		
sed	sed-Fw	CAAATATATTGATATAATGA	330	[12]
	sed-Rv	AGTAAAAAAGAGTAATGCAA		
see	see-Fw	CAAAGAAATGCTTTAAGCAATCTTAGGC	482	[12]
	see-Rv	CACCTTACCGCCAAAGCTG		
tst	tst-Fw	ACCCCTGTTCCCTTATCATC	326	[12]
	tst-Rv	TTTTCAGTATTTGTAACGCC		
lukF-lukS	luk-PV-Fw	ATCATTAGGTAAAATGTCTGGACATGATCCA	433	[13]
	luk-PV-Rv	GCATCAASTGTATTGGATAGCAAAAGC		
spa **typing**				
spa	spa-1113-Fw	TAAAGACGATCCTTCGGTGAGC	110-442	[14]
	spa-1514-Rv	CAGCAGTAGTGCCGTTTGCTT		

bp: base pair; **Fw:** "forward"; **Rv:** "reverse

Table S1. Primers used in this study

Target Gene	Primers	Nucleotide Sequence (5′-3′)	Amplicon Size (bp)	Reference
		**MLST**		
arcC	arcC_Fw	TTGATTCACCAGCGCGTATTGTC	456	[15]
	arcC_Rv	AGGTATCTGCTTCAATCAGCG		
aroE	aroE_Fw	ATCGGAAATCCTATTTCACATTC	456	[15]
	aroE_Rv	GGTGTTGTATTAATAACGATATC		
glpF	glpF_Fw	CTAGGAACTGCAATCTTAATCC	465	[15]
	glpF_Rv	TGGTAAAATCGCATGTCCAATTC		
gmk	gmk_Fw	ATCGTTTTATCGGGACCATC	429	[15]
	gmk_Rv	TCATTAACTACAACGTAATCGTA		
pta	pta_Fw	GTTAAAATCGTATTACCTGAAGG	474	[15]
	pta_Rv	GACCCTTTTGTTGAAAAGCTTAA		
tpi	tpi_Fw	TCGTTCATTCTGAACGTCGTGAA	402	[15]
	tpi_Rv	TTTGCACCTTCTAACAATTGTAC		
yqiL	yqiL_Fw	CAGCATACAGGACACCTATTGGC	516	[15]
	yqiL_Rv	CGTTGAGGAATCGATACTGGAAC		

bp: base pair; **Fw:** "forward"; **Rv:** "reverse

References

1. Brakstad, O.G.; Aasbakk, K.; Maeland, J.A. Detection of Staphylococcus Aureus by Polymerase Chain Reaction Amplification of the Nuc Gene. *J. Clin. Microbiol.* **1992**, *30*, 1654–1660.
2. Petinaki, E. Detection of MecA, MecR1 and MecI Genes among Clinical Isolates of Methicillin-Resistant Staphylococci by Combined Polymerase Chain Reactions. *Journal of Antimicrobial Chemotherapy* **2001**, *47*, 297–304, doi:10.1093/jac/47.3.297.
3. Milheiriço, C.; Portelinha, A.; Krippahl, L.; de Lencastre, H.; Oliveira, D.C. Evidence for a Purifying Selection Acting on the β-Lactamase Locus in Epidemic Clones of Methicillin-Resistant Staphylococcus Aureus. *BMC Microbiol* **2011**, *11*, 76, doi:10.1186/1471-2180-11-76.
4. Jensen, L.B.; Hammerum, A.M.; Bager, F.; Aarestrup, F.M. Streptogramin Resistance among *Enterococcus Faecium* Isolated from Production Animals in Denmark in 1997. *Microbial Drug Resistance* **2002**, *8*, 369–374, doi:10.1089/10766290260469642.
5. Costa, S.S.; Palma, C.; Kadlec, K.; Fessler, A.T.; Viveiros, M.; Melo-Cristino, J.; Schwarz, S.; Couto, I. Plasmid-Borne Antimicrobial Resistance of *Staphylococcus Aureus* Isolated in a Hospital in Lisbon, Portugal. *Microbial Drug Resistance* **2016**, *22*, 617–626, doi:10.1089/mdr.2015.0352.
6. Ferreira, C.; Costa, S.S.; Serrano, M.; Oliveira, K.; Trigueiro, G.; Pomba, C.; Couto, I. Clonal Lineages, Antimicrobial Resistance, and PVL Carriage of Staphylococcus Aureus Associated to Skin and Soft-Tissue Infections from Ambulatory Patients in Portugal. *Antibiotics* **2021**, *10*, 345, doi:10.3390/antibiotics10040345.
7. Strommenger, B.; Kettlitz, C.; Werner, G.; Witte, W. Multiplex PCR Assay for Simultaneous Detection of Nine Clinically Relevant Antibiotic Resistance Genes in *Staphylococcus Aureus*. *J Clin Microbiol* **2003**, *41*, 4089–4094, doi:10.1128/JCM.41.9.4089-4094.2003.
8. Aarestrup, F.M.; Agerso, Y.; Gerner–Smidt, P.; Madsen, M.; Jensen, L.B. Comparison of Antimicrobial Resistance Phenotypes and Resistance Genes in Enterococcus Faecalis and Enterococcus Faecium from

Humans in the Community, Broilers, and Pigs in Denmark. *Diagnostic Microbiology and Infectious Disease* **2000**, *37*, 127–137, doi:10.1016/S0732-8893(00)00130-9.
9. Costa, S.S.; Oliveira, V.; Serrano, M.; Pomba, C.; Couto, I. Phenotypic and Molecular Traits of Staphylococcus Coagulans Associated with Canine Skin Infections in Portugal. *Antibiotics* **2021**, *10*, 518, doi:10.3390/antibiotics10050518.
10. Couto, N.; Belas, A.; Couto, I.; Perreten, V.; Pomba, C. Genetic Relatedness, Antimicrobial and Biocide Susceptibility Comparative Analysis of Methicillin-Resistant and -Susceptible *Staphylococcus Pseudintermedius* from Portugal. *Microbial Drug Resistance* **2014**, *20*, 364–371, doi:10.1089/mdr.2013.0043.
11. Argudín, M.A.; Tenhagen, B.-A.; Fetsch, A.; Sachsenröder, J.; Käsbohrer, A.; Schroeter, A.; Hammerl, J.A.; Hertwig, S.; Helmuth, R.; Bräunig, J.; et al. Virulence and Resistance Determinants of German Staphylococcus Aureus ST398 Isolates from Nonhuman Sources. *Appl. Environ. Microbiol.* **2011**, *77*, 3052–3060, doi:10.1128/AEM.02260-10.
12. Wang, W.; Lin, X.; Jiang, T.; Peng, Z.; Xu, J.; Yi, L.; Li, F.; Fanning, S.; Baloch, Z. Prevalence and Characterization of Staphylococcus Aureus Cultured From Raw Milk Taken From Dairy Cows With Mastitis in Beijing, China. *Front. Microbiol.* **2018**, *9*, 1123, doi:10.3389/fmicb.2018.01123.
13. Lina, G.; Piemont, Y.; Godail-Gamot, F.; Bes, M.; Peter, M.-O.; Gauduchon, V.; Vandenesch, F.; Etienne, J. Involvement of Panton-Valentine Leukocidin--Producing Staphylococcus Aureus in Primary Skin Infections and Pneumonia. *Clinical Infectious Diseases* **1999**, *29*, 1128–1132, doi:10.1086/313461.
14. Aires-de-Sousa, M.; Boye, K.; de Lencastre, H.; Deplano, A.; Enright, M.C.; Etienne, J.; Friedrich, A.; Harmsen, D.; Holmes, A.; Huijsdens, X.W.; et al. High Interlaboratory Reproducibility of DNA Sequence-Based Typing of Bacteria in a Multicenter Study. *Journal of clinical microbiology* **2006**, *44*, 619–21.
15. Enright, M.C.; Day, N.P.; Davies, C.E.; Peacock, S.J.; Spratt, B.G. Multilocus Sequence Typing for Characterization of Methicillin-Resistant and Methicillin-Susceptible Clones of Staphylococcus Aureus. *Journal of Clinical Microbiology* **2000**, *38*, 1008–15.
